# Solvent Vapor Annealing for Controlled Pore Expansion of Block Copolymer-Assembled Inorganic Mesoporous Films

**DOI:** 10.1021/acs.langmuir.2c00074

**Published:** 2022-03-02

**Authors:** Alberto Alvarez-Fernandez, Maximiliano Jara Fornerod, Barry Reid, Stefan Guldin

**Affiliations:** Department of Chemical Engineering, University College London, Torrington Place, London WC1E 7JE, U.K.

## Abstract

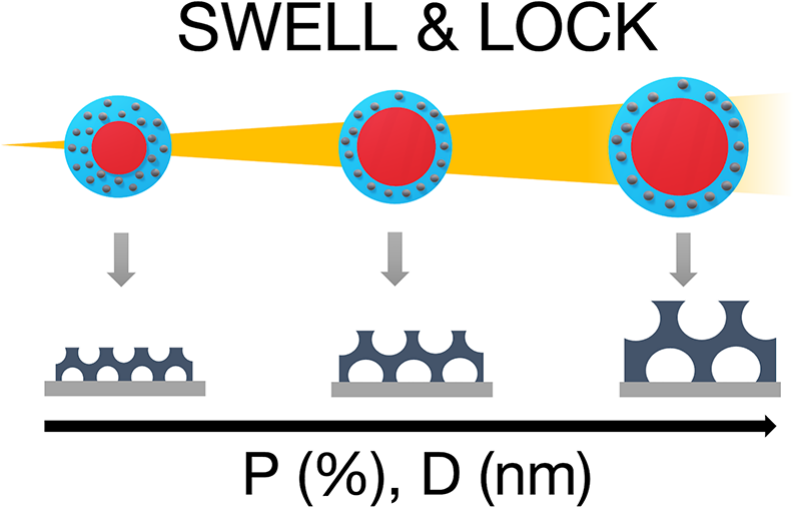

Mesoporous inorganic thin films are promising materials architectures for a variety of high-value applications, ranging from optical coatings and purification membranes to sensing and energy storage devices. Having precise control over the structural parameters of the porous network is crucial for broadening their applicability. To this end, the use of block copolymers (BCP) as sacrificial structure-directing agents via micelle coassembly is a particularly attractive route, since the resultant pore size is directly related to scaling laws for the radius of gyration of the pore-forming macromolecule. However, tailoring the molecular weight of the BCP via bespoke synthesis is an elaborate process that requires precise control over highly sensitive reactions conditions. Alternative methods have emerged, based on supramolecular assembly or the addition of different swelling agents. Nevertheleses, to date, these present a negative impact on the structural order and pore size dispersity of the final inorganic mesoporous films. In this work, we propose a novel and effective method for control over pore size, porosity, and structural order, which relies on a synergistic combination of BCP selective swelling via solvent vapor annealing (SVA) and locking of the structure by condensation of the inorganic sol–gel precursors. The results obtained in this work for TiO_2_ establish SVA as a new, straightforward, simple, and powerful route for the fabrication of mesoporous thin-film materials with controllable structural characteristics.

## Introduction

Mesoporous architectures with pores on the 5–50 nm length scale offer distinct opportunities for a wide range of applications, such as energy conversion and storage devices,^1−3^ separation and purification membranes,^4,5^ chemical/biosensors,^6,7^ or optical coatings.^8,9^ Having precise control over the mesoporous structural parameters, i.e., pore size, pore arrangement, and overall porosity, constitutes in many use cases an important requirement.^10,11^

Bottom-up fabrication strategies based on the use of sacrificial structure-directing agents (SDAs) have proven a particularly attractive method to create ordered mesoporous thin films with tunable pore size and porosity.^12−15^ Following this approach, SDAs interact with inorganic precursors (typically sol–gel derived) via preferential supramolecular interactions in solution. In a subsequent step, hybrid composites are produced via evaporation-induced coassembly and transformed into an ordered inverse opal-type mesoporous structure by thermal calcination or other chemical degradation processes.^16,17^ While small surfactant molecules are suitable sacrificial blocks for the fabrication of 2–5 nm pore size mesoporous structures,^18,19^ the use of block copolymers (BCPs) as SDAs constitutes a versatile, straightforward, cost-effective, and reliable method for the fabrication of larger pore size architectures (8–50 nm).^20−22^ In the case of BCP coassembly, the final mesoporous structure can be easily tuned by controlling the macromolecular characteristics of the starting BCP, i.e., the degree of polymerization (N) and mixing ratio between BCP and inorganic precursors. While pore size is commonly determined by the molecular weight of the pore-forming segment of the BCP, control over the mixing ratio allows the fine-tuning of the total porosity of the sample.^23−25^ Therefore, the synthesis of BCPs with well-defined molecular weight for each of the blocks is imperative for precise control over the resulting mesoporous films. However, the tailored synthesis of BCP constitutes a challenging and elaborate process that often involves multiple purification steps, precise reaction conditions, and controlled atmosphere procedures, limiting overall the implementation of this approach as a standard method for tailoring the pore size.^26,27^

Extensive research has been performed in the past decade in the search for alternative and complementary methods for limiting the synthetic effort necessary for continuous pore tuning and nanostructure optimization. One approach introduced by our group is size exclusion chromatographic fractionation of polydisperse BCPs, which may serve for systematic pore size control and reduction of dispersity of the resulting mesoporous inorganic thin film architectures.^28^ Alternative methods based on pore expansion by supramolecular coassembly of swelling agents, carefully chosen to selectively interact with the pore-forming block, have been successfully implemented. To this end, benzene derivatives,^29,30^ homopolymers,^31,32^ carboxylic acids,^33,34^ or solvents such as toluene or xylene^35,36^ can be used for tuning structural dimensions of the final inorganic mesoporous thin film. However, following this approach, and contrary to the size exclusion chromatography, a negative impact in pore size dispersity and long-range order of the structure has been identified.^37^ Moreover, macroscopic phase separation at large swelling agent BCP ratios limits the application range. Therefore, the search for simple, fast, and scalable approaches that allow for continuous pore tuning remains a challenging research endeavor.

To this end, solvent vapor annealing (SVA) constitutes an interesting approach. SVA is a widely used technique in the BCP nanolithography field for controlling both the final BCP morphology and the microstructure orientation in thin-film configuration.^38−40^ During SVA treatments, BCP thin films are exposed to vapors of one or more solvents that swell the film and provide mobility to the polymer chains to diffuse and reorganize, promoting the ordering of the BCP structures. However, after a rapid dry quenching, films typically recover their original thickness.^41,42^

In this work, we propose the combination of solvent vapor annealing (SVA) and sol–gel reaction as a promising and effective alternative method for pore expansion on mesoporous thin films. To this end, hybrid films composed of the amphiphilic block copolymer poly(isobutylene)-*block*-poly(ethylene oxide) (PIB-*b*-PEO) and TiO_2_ sol precursors are swollen by exposing samples to suitable solvent vapors that selectively swell the PIB block for a defined amount of time. Concurrently, the swollen structure is locked in place by the condensation reaction of the inorganic precursors, which is followed by Fourier-transform infrared spectroscopy (FTIR). Finally, the mesoporous TiO_2_ thin films are characterized by scanning electron microscopy (SEM), atomic force microscopy (AFM), and ellipsometric porosimetry (EP) to fully validate this approach.

## Results

**Figure 1:**
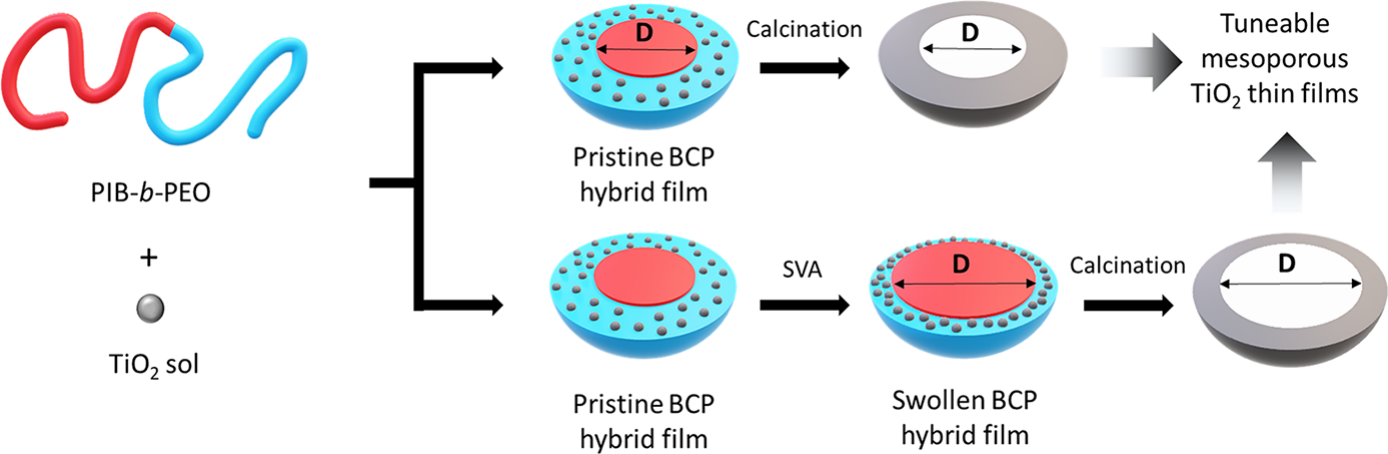
Schematic illustration of the BCP coassembly swell and lock SVA pore expansion process. Hybrid BCP films are exposed to cyclohexane vapors, with the consequent swelling of the micelle PIB core. After 30 min or 1 h at room temperature, samples are removed and calcined at 450 °C. The partial condensation reaction of the inorganic sol–gel precursors during the SVA process allows tuning the pore size and porosity of the final inorganic structure.

Tunable mesoporous structures were obtained following the methodology sketched in Figure 1. The pore-forming polymer block, PIB, is swollen by exposing samples to vapors of a selective solvent. Concurrently, the structure is locked in place by the condensation reaction of the inorganic precursors, allowing to have a precise pore size tuning via pore swelling.

The first critical step is therefore to choose the correct solvent to have a selective swelling of the pore-forming block. Table 1 lists the respective polymer–solvent interaction parameters, calculated by using the Hansen solubility values for both BCP blocks (PIB and PEO) and common organic solvents (THF, cyclohexane, and toluene) studied during this work.

**Table 1:** Hansen Solubility Parameters for the BCP Materials Studied

solvent or polymer	δ_*d*_ (MPa^1/2^)	δ_p_ (MPa^1/2^)	δ_*h*_ (MPa^1/2^)	*V* (μm^3^ mol^–1^)	*V*_*p*_^a^ (kPa)	χ_pol-THF_^b^	χ_pol-cyclohexane_^b^	χ_pol-toluene_^b^
THF^43^	16.8	5.7	8.0	81.7	23.4			
cyclohexane^44^	16.8	0.00	*0*.20	108.9	13.1			
toluene^43^	18.0	1.4	2.0	137.1	3.8			
PIB^45^	14.5	2.07	4.66	63.3		0.29	0.28	0.36
PEO^46^	17.3	3.0	9.4	38.9		0.04	0.37	0.23

a Vapor pressures are at 298 K.

b Values of χ parameters are estimated at 298 K by using χ_*ij*_ = (*V*/*RT*)[(δ_*dj*_ – δ_*di*_)^2^ + 0.25(δ_*pj*_ – δ_*pi*_)^2^ + 0.25(δ_*hj*_ – δ_*hi*_)^2^] , where δ_*d*_, δ_*p*_, and δ_*h*_ are parameters related to dispersion, polarity, and hydrogen bonding, respectively, *V* is the molar volume, and χ is the Flory–Huggins interaction parameter.

From a consideration of interaction parameters, all screened solvents are suitable solvents for swelling the PIB block (χ_PIB-solvent_ < 0.4). However, THF exhibits a higher affinity for the PEO block (χ_PIB-THF_ = 0.29 vs χ_PEO-THF_ = 0.04), making it less appropriate for the selective swelling of the PIB micelle core. In this sense, cyclohexane offers more suitable characteristics, since it presents a low interaction parameter with the PIB part while displaying a high interaction parameter with the PEO block, allowing the selective swelling of the former block.

To gain further insights into the BCP film swelling process, a pure PIB-*b*-PEO film was deposited on a Si substrate by spin-coating and enclosed in a solvent annealing chamber. Vapors of the three different solvents were consecutively introduced, and the film thickness was recorded in situ by ellipsometry. Figure S1 shows the film swelling after 25 min of SVA for each solvent. In all cases, film thickness increased from the initial 116 nm to 136, 150, and 155 nm by using toluene, cyclohexane, or THF, respectively. The lower vapor pressure of toluene compared with THF or cyclohexane explains its low swelling ratio, while the low selectivity of the THF is in line with the higher swelling ratios observed. Therefore, and taking into account its low vapor pressure, high selectivity, and high swelling ratios, cyclohexane emerged as the most suitable solvent for selective PIB-*b*-PEO swelling and was used during the subsequent SVA experiments.

**Figure 2:**
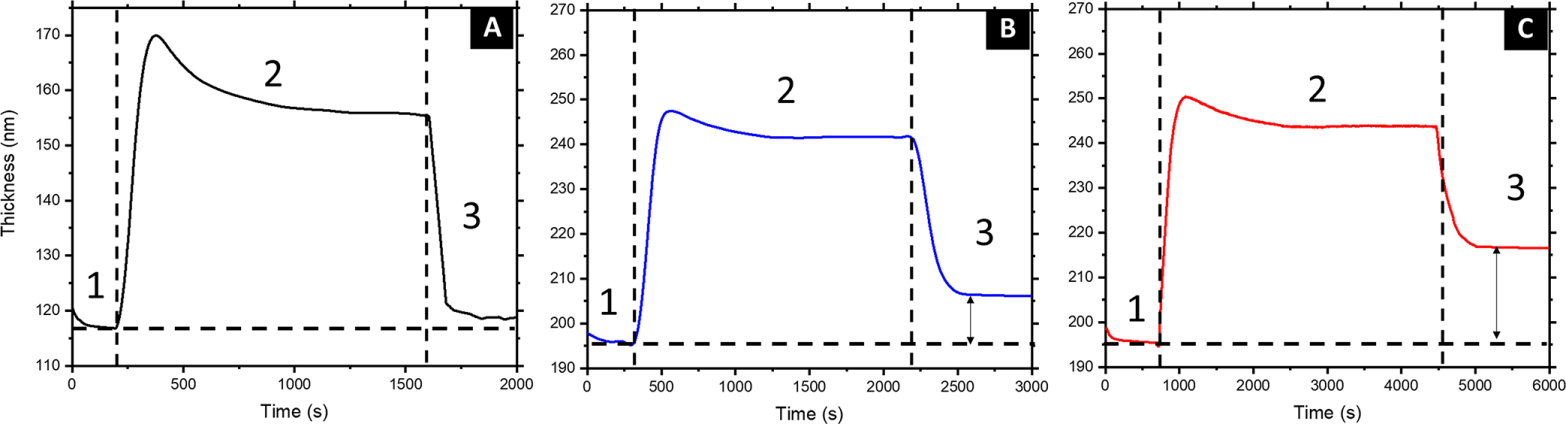
Film thickness evolution profile during the SVA treatment for a pure BCP (25 min) (A) and a hybrid BCP:TiO_2_ sol thin film (B, 30 min; C, 1 h).

To study the swelling behavior of the coassembled organic–inorganic film, a hybrid BCP:TiO_2_ sol solution with a O:I ratio corresponding to α (see the Experimental Section) was deposited onto a silicon wafer and introduced in the SVA chamber. SVA experiments were followed in situ by ellipsometry to monitor the swelling process (Figure S2). In a first step (1 in Figure 2) N_2_ gas was introduced in the chamber to stabilize film thickness. After cyclohexane entered the chamber (2 in Figure 2), the hybrid film started to swell, reaching a maximum thickness of 245–250 nm (from the original 195 nm), which was kept constant during the duration of the SVA treatment. Finally, pure N_2_ gas was introduced again in the chamber (3 in Figure 2), invoking the film to deswell. In contrast to purely organic BCP films (Figure 2A), in which the original thickness was recovered after solvent removal, hybrid BCP:TiO_2_ sol films were able to partially retain their swollen thickness (Figure 2B). Interestingly, longer solvent annealing of the hybrid films led to a higher overall thickness at the end of the process (Figure 2C).

**Figure 3:**
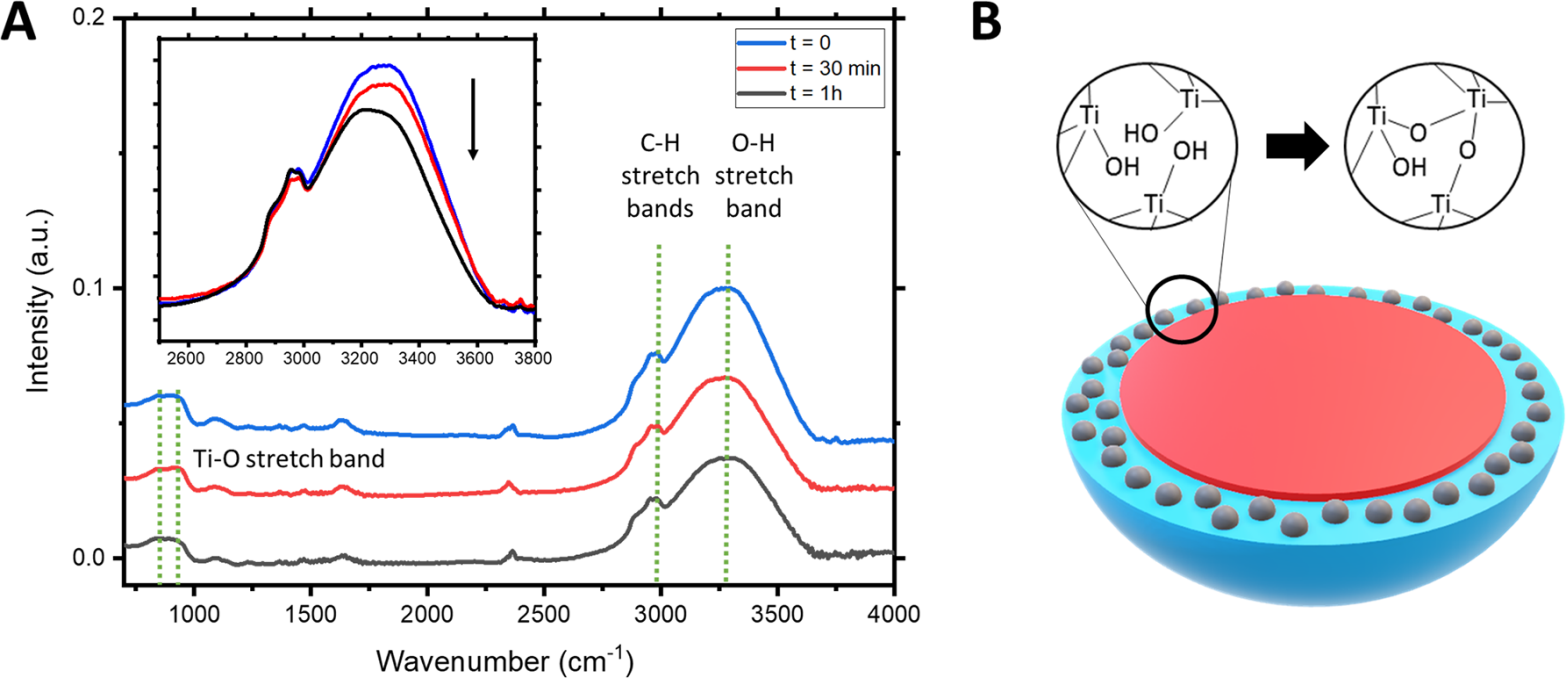
(A) FTIR spectra of the hybrid BCP samples before (black line) and after (red and blue lines) SVA. (B) Schematic representation of the partial condensation reaction that takes place in the shell of the micelle during the SVA process.

The different behavior observed between pure and hybrid BCP film may be explained by taking into account the sol–gel reaction of the inorganic precursors presented in the BCP hybrid micelles. Thus, FTIR measurements were performed at different stages of the process to monitor the condensation reaction. Figure 3A shows the FTIR spectra of the hybrid samples (i.e., before final calcination) at different times: *t* = 0 min (reference) and after 30 min and 1 h SVA. All spectra present similar bands: a broad band around 3250 cm^–1^, which is attributed to the O–H stretching mode of Ti–OH groups, a sharp peak centered around 2800 cm^–1^ corresponding to the C–H stretch of the polymeric chains, and a band at 800 cm^–1^ due to the Ti–O bond stretching mode.

Interestingly, a direct comparison of all spectra in the 2500–3800 cm^–1^ area (see the inset in Figure 3A) shows a clear reduction in the O–H band intensity during the SVA, suggesting a partial condensation reaction of the Ti–OH sol precursors located at the micelle shell (Figure 3B). This partial reaction is due to the spontaneous condensation of the hydroxo-Ti complex formed during the hydrolysis of the Ti precursors.^47^ In the case of the BCP-Ti hybrid films, this condensation reaction, even if not completed, provided enough mechanical strength to the swollen structure to retain in an expanded state after the sample is removed from the SVA chamber (Figure 2B,C). In contrast, when pure BCP film, i.e., with no inorganic sol, was exposed to the SVA, the polymer film recovered its original thickness once removed from the chamber, as previously reported for BCP SVA systems.^48^

**Figure 4:**
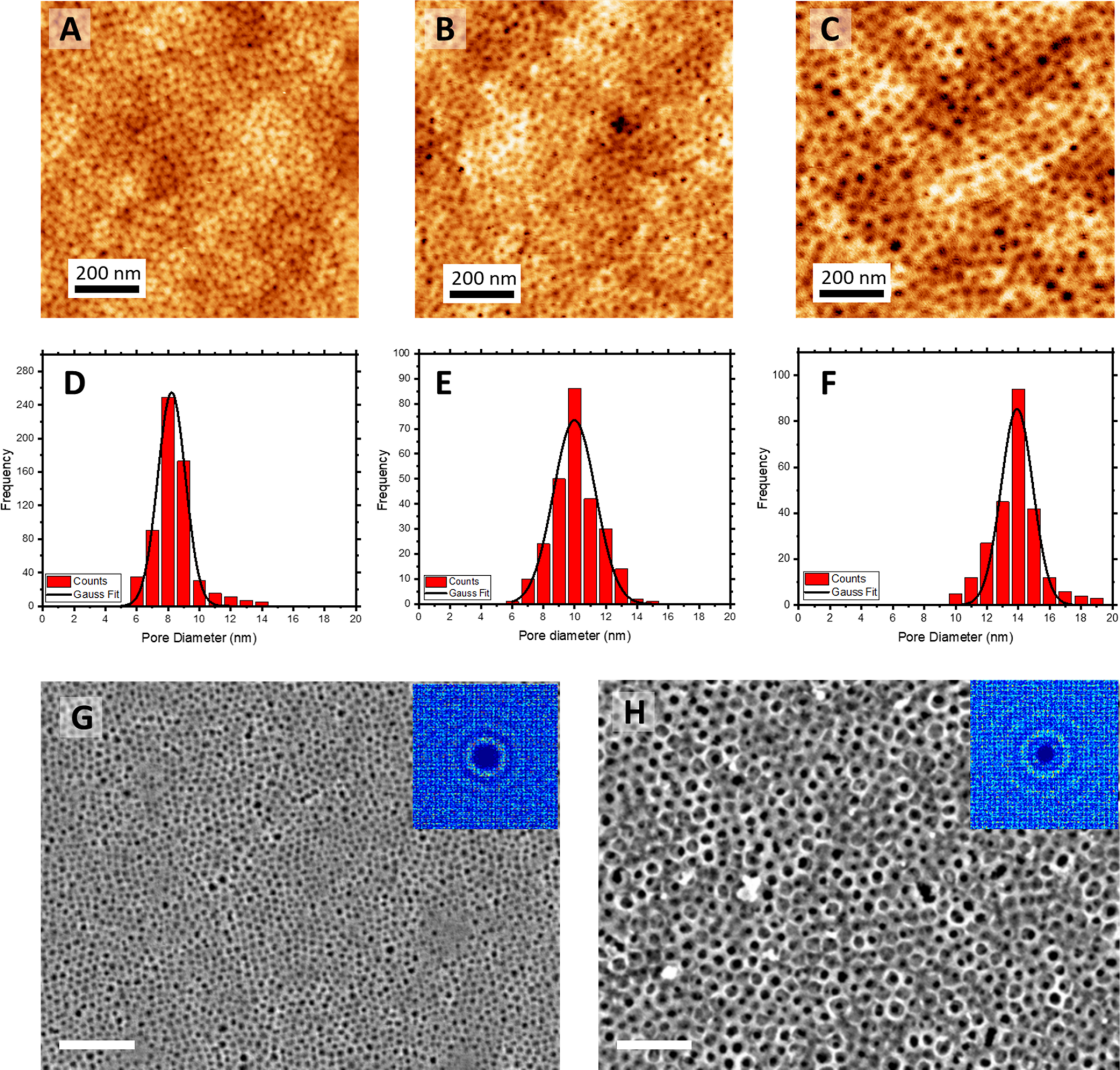
AFM topographical images of the TiO_2_ mesoporous films (α, α*) obtained with no SVA (A) and after 30 min (B) and 1 h (C) SVA treatment. Corresponding pore diameter histograms obtained by image analysis (D–F). SEM images of the TiO_2_ mesoporous films with no SVA (G) and after 1 h SVA treatment (H). The inset corresponds to the 2D spatial distribution function to evaluate pore ordering. Scale bar: 150 nm.

To study the effect of the SVA in the final inorganic mesoporous structure, hybrid samples were calcined in a furnace for the complete removal of the BCP. Figure 4A–C shows a comparison of the topographical AFM micrographs for the corresponding mesoporous TiO_2_ films. A clear enlargement of the pore structure can be detected as a result of the selective film swelling and structural locking during the SVA treatment. To gain more insights into the porous characterization of the mesoporous architectures, the average pore diameter was determined by analysis of the real space topography images using the software Pebbles (Figure S3). Pore size distribution histograms show a clear evolution of the structure during the annealing process with pore size diameter (*D*) increasing from *D* = 8.2 ± 1.7 nm (*t* = 0) to *D* = 9.9 ± 2.5 nm (*t* = 30 min) and *D* = 13.9 ± 2.0 nm (*t* = 1 h) (Figure 4D–F). It is important to mention that longer annealing times (1.5 h) did not result in a continuation of this trend. As presented in Figure S4, AFM topographical micrography showed a gradual loss of the highly ordered structure. While the majority of pores displayed diameters of around 15 nm, larger pores with *D* > 50 nm started appearing, indicating polymer rearrangement and reconstruction of the micelle packing, an unwanted effect in this context that enhanced pore dispersity. Therefore, SVA annealing times were limited in the following to 1 h.

As previously introduced, one major limitation of swelling agents to date is their negative impact on the structural order and pore size dispersity of the final inorganic films. Crucially, swelling by SVA can achieve the opposite effect, namely an improvement of structural order. Figure 4G,H shows the SEM micrographs of the mesoporous TiO_2_ films obtained with and without SVA treatment. In line with previous AFM measurements, a clear enlargement of the porous structure can be detected. SEM micrographs were analyzed by using CORDERLY^49^ (an alternative software to the standard 2D fast Fourier transform (FFT)) to evaluate the impact of the SVA treatment on the mesoporous arrangement of the samples. The higher number of concentric hexagonal rings displayed in the 2D spatial distribution function (SDF) (insets in Figure 4G,H) of the sample exposed to the SVA treatment suggests a higher degree of order compared to the nontreated sample, which provides a further advantage of the approach.

To confirm the critical role of the sol–gel condensation reaction in the process to lock the structure, an alternative sol with lower reactivity was also explored during this work. Hybrid BCP–aluminosilicate sol films were prepared and exposed to the same SVA experimental conditions as TiO_2_ films. Figure S5A,B shows the AFM micrographs of the aluminosilicate mesoporous films obtained after calcination. While an improvement in the structural order was also found in this case, no clear impact in the pore size distribution or porosity was observed (Figure S5C,D). While for previous TiO_2_ hybrid films a partial spontaneous condensation reaction was detected, FTIR spectra of the hybrid aluminosilicate films present identical intensities before and after the SVA treatment (Figure S5E). The absence of spontaneous condensation reaction prevented any pore expansion effect. These results highlight the importance of the sol–gel condensation reaction in providing the necessary structural integrity to the hybrid film to retain a pore enlargement after SVA.

**Figure 5:**
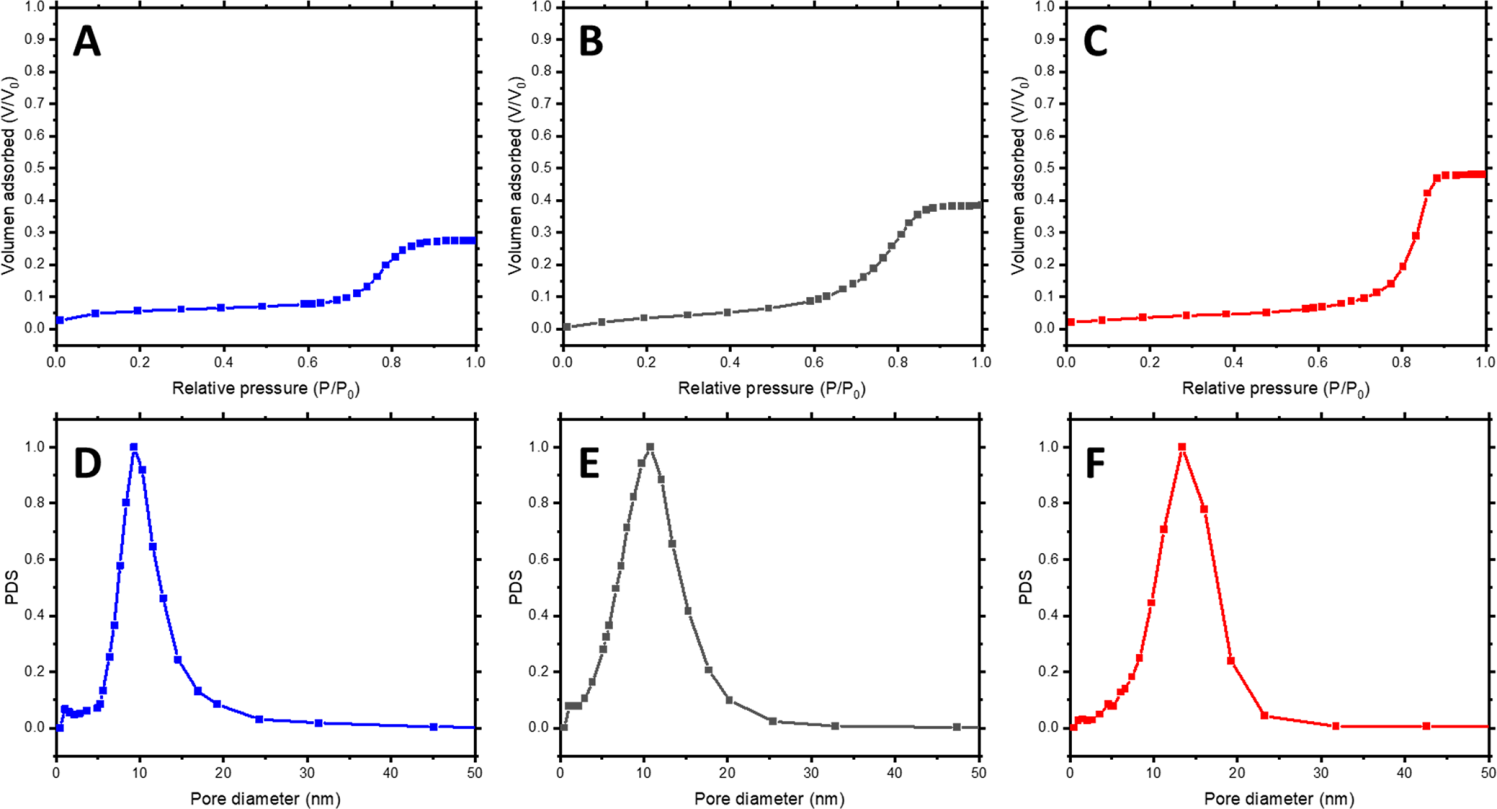
EP adsorption isotherms (A–C) with correlated pore size distributions (D–F) for the nontreated film (A, D), 30 min cyclohexane SVA (B, E), and 1 h cyclohexane SVA (C, F).

Previous works have put AFM and SEM in context to other techniques for a precise structural characterization of mesoporous thin films, showing their limitations in providing essential structural information such as out-of-plane pore dimensions and spacing or porosity.^20^ Thus, for a more complete view, samples were also analyzed by ellipsometric porosimetry (EP). Figure 5A–C shows the adsorption isotherm for the studied samples. A clear change in the total porosity of the sample was observed with the SVA treatment (29 vs 48% after 1 h SVA in cyclohexane). Moreover, further analysis of the EP adsorption isotherms using the Kelvin equation allowed obtaining the pore radius distribution of the mesoporous thin films. While untreated samples (α) exhibited a pore size diameter *D* = 8.9 ± 1.5 nm (Figure 5D), identical samples exposed 1 h to cyclohexane vapor (α*) presented a *D* = 14.8 ± 1.9 nm (Figure 5F). These results confirm the controlled expansion of the inorganic structure after the swelling and locking SVA procedure. An intermediate result with *D* = 10.3 ± 2.1 nm (Figure 5B,E) was observed for a shorter SVA treatment (30 min), demonstrating the possibility of tuning the structural parameters by the length of the SVA treatment.

**Figure 6:**
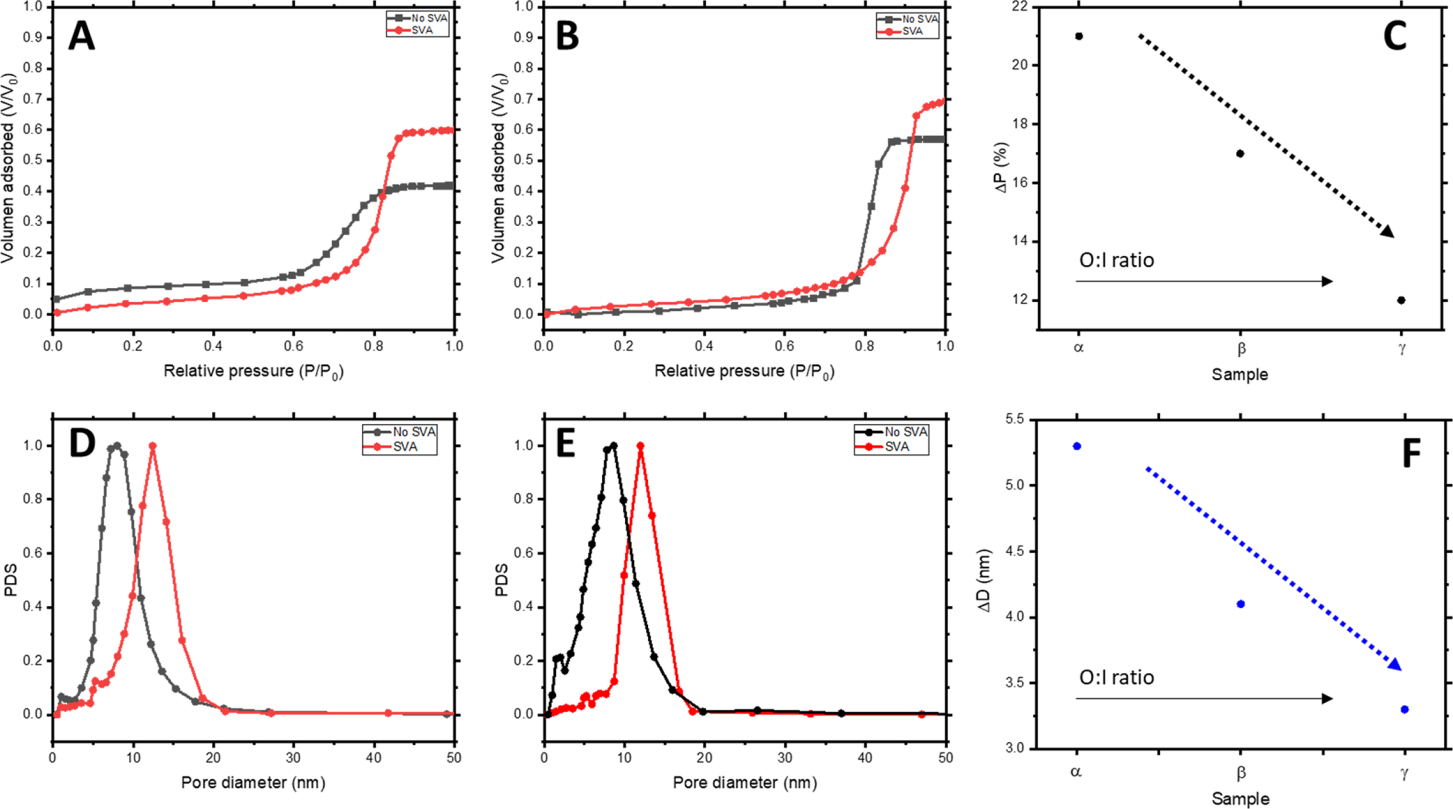
EP adsorption isotherms (top) with correlated pore size distributions (bottom) for different O:I ratios denoted as β (A, D) and γ (B, E) each with (red line) and without (black line) SVA. Effect of the SVA process over porosity (C) and pore diameter (F) for the three different O:I ratios presented in this work (α, β, and γ).

To study the effect of the inorganic content in the final structure obtained after SVA, samples with increasing O:I ratios were exposed 1 h to cyclohexane vapors. Figures 6A and 6B show the EP adsorption isotherms for β and γ samples, respectively. In both cases, the increment in the organic content in the starting hybrid solution allows increasing the original porosity of nontreated samples (42% and 58%, respectively) compared with the 29% observed for α (Figure 5A). This is in line with previous observations where total porosity values were controlled by the O:I ratio.^12^ We consistently measured a higher porosity after the SVA for films regardless of their O:I ratio. Thus, final porosity retained for β* samples increased from 42% to 59% and for γ* from 58% to 70% with the SVA (Figure 6A,B).

Nontreated α, β, and γ samples presented very similar pore sizes, with values ranging from 8.9 ± 1.5 nm to 8.3 ± 1.6 and 8.6 ± 1.8 nm, respectively. However, a notable difference in pore sizes was observed after the SVA treatment. The sample with the lower O:I ratio (α*) increased the pore size from *D* = 8.9 ± 1.5 to *D* = 14.8 ± 1.9 nm with the SVA, as previously discussed. Increasing the O:I ratio (β*) led to a pore size of *D* = 12.4 ± 2.0 nm, while *D* values for higher O:I ratio (γ*) reached *D* = 11.9 ± 1.9 nm. Apart from the clear pore size increment as a consequence of the SVA, another tendency can be extracted with the systematic change in the O:I ratio: the ability of the condensation reaction to lock the swollen structure is proportional to the inorganic content in the hybrid solution (Figure 6C,F). We relate this trend to the higher structural integrity of the partially condensed Ti network obtained with higher inorganic content.

## Conclusion

In this work, we establish solvent vapor annealing (SVA) as a new and straightforward approach for tuning the pore size and porosity in TiO_2_ mesoporous thin film architectures fabricated by the coassembly of block copolymers with sol–gel precursors. The approach relies on a synergistic interplay between dimensional tuning by selective solvent swelling of the pore-forming BCP block and the spontaneous condensation of the inorganic matrix. A complete library of TiO_2_ mesoporous coatings with different porosity and pore sizes was obtained by exposing organic–inorganic hybrid films to cyclohexane vapors for 30 min and 1 h. The combination of structural characterization by AFM, SEM, and EP and the chemical information obtained by FTIR allowed the establishment of a close relationship between the sol–gel condensation reaction and the final expansion of the mesoporous structure obtained after SVA. Moreover, a clear enhancement in the long-range order of the final inorganic mesoporous structure was found as a result of the SVA treatment. These results highlight the relevance and versatility of the SVA as a new standard method for the controllable expansion of mesoporous thin films.

## Experimental Section

### Reagents

PIB_3.9_-*b*-PEO_3.6_ block copolymer (polydispersity 1.26, *M*_n_ 4.85 kg mol^–1^) was provided by BASF. Toluene (99.9%), 1-butanol (99.4%), hydrochloric acid (HCl, fuming 37%), and titanium isopropoxide (TiOPr, >98%) were purchased from Merck. All chemicals were used without further purification.

### Preparation of Mesoporous TiO_2_ Films

PIB_3.9_-*b*-PEO_3.6_ BCP (*M*_n_ 4.85 kg/mol; polydispersity index (PDI) 1.26) was supplied by BASF following a previously reported synthetic route.^50^ Inorganic sol material and mesoporous inorganic TiO_2_ films were prepared as described in previous works.^8,16^ Thus, a 50 mg/mL BCP solution was prepared by using toluene/1-butanol azeotrope (72.84/27.16 wt %) as solvent. The TiO_2_ inorganic sol was prepared by the addition of 0.193 mL of TiOPr to 0.061 mL of HCl under continuous stirring. In a final step different organic–inorganic (O:I) ratio solutions were prepared as described in Table 2. All samples were deposited by spin-coating on 1 × 1 cm^2^ silicon substrates at 2000 rpm for 30 s. Immediately after the film deposition, samples were introduced in the solvent vapor annealing (SVA) chamber (Figure S2A). The solvent-rich atmosphere inside the chamber was created by the continuous stream of a toluene, THF, or cyclohexane vapors produced by bubbling nitrogen gas (0.1 L min^–1^, flow controller F-201CV, Bronkhorst) through the corresponding liquid solvent. SVA treatments were performed for up to 1 h at room temperature (22 °C). Finally, and to remove the BCP, samples were calcined in a muffle furnace at 450 °C for 30 min.

**Table 2:** List of Samples That Were Studied during This Work

sample	vol of TiO_2_ (mL)	vol of HCl (mL)	BCP solution vol (mL)
α/α^a^	0.193	0.061	0.650
β/β^a^	0.193	0.061	1
γ/γ^a^	0.193	0.061	1.250

a SVA sample.

### Samples Characterization

AFM images were obtained on a Bruker Dimension Icon atomic force microscope with a Bruker ScanAsyst Air probe (nominal tip radius 2 nm) in tapping mode. The average pore radius was determined by analysis of the real space topography images using the Pebbles software.^51^

SEM images were taken in a Xbeam 540 FIB/SEM (ZEISS) directly on TiO_2_ mesoporous films without any metallic coating. Images were captured by using an acceleration voltage between 0.5 and 2 kV and working distance between 0.9 and 1 mm. The 2D spatial distribution function was calculated with the software CORDERLY.^49^

Ellipsometric porosimetry (EP) measurements were performed on a Semilab SE2000 variable angle spectroscopic ellipsometer in the spectral range 300–1000 nm. All data analysis was performed by using the Semilabs SEA software (v1.6.2). Before EP measurements, samples were placed on a hot plate at 120 °C for 10 min to remove residual atmospheric water molecules inside the pores. Fourier-transform infrared spectroscopy (FTIR) measurements of the samples before and after the SVA step were performed by using a AIM-9000 infrared microscope coupled with IRTracer-1000 FTIR spectrophotometer (Shimadzu) in reflection mode. Atmospheric and baseline correction were performed with the software Lab Solutions IR (Shimadzu). To increase the sample reflectance, gold-coated Si substrates were used during this study.

## Supporting Information Available

In-situ ellipsometry of film swelling for purely organic block copolymer samples in various solvents; information on the experimental setup used for SVA; evidence of the image analysis used for pore size determination; AFM micrograph of mesoporous film after 1.5 h of SVA; experimental data (AFM, ellipsometric porosimetry, FTIR) for aluminosilicate samples (PDF)
