# Supplementary material for: Solvent Vapor Annealing for Controlled Pore Expansion of Block Copolymer-Assembled Inorganic Mesoporous Films
Source: Langmuir. 2022 Mar 2;38(10):3297–304. doi: 10.1021/acs.langmuir.2c00074 (PMC9097528; doi:10.1021/acs.langmuir.2c00074)
Supplement: Supplementary file 1 — la2c00074_si_001.pdf [file la2c00074_si_001.pdf]

# - Supporting Information-

Langmuir, DOI: 10.1021/acs.langmuir.2c00074

Solvent vapor annealing for controlled pore expansion of block copolymer-assembled inorganic mesoporous films

Alberto Alvarez-Fernandez,[†] *Maximiliano Jara Fornerod,*[†] *Barry Reid,*[†] *and Stefan Guldin*[†, *]

[†]Department of Chemical Engineering, University College London, Torrington Place, London, WC1E 7JE, UK

E-mail: s.guldin@ucl.ac.uk

Supporting Information content:

- In-situ ellipsometry of film swelling for purely organic block copolymer samples in various solvents.
- Information of the experimental set-up used for SVA.
- Evidence of the image analysis used for pore size determination.
- AFM micrograph of mesoporous film after 1.5h of SVA.
- Experimental data (AFM, ellipsometric porosimetry, FTIR) for aluminosilicate samples.

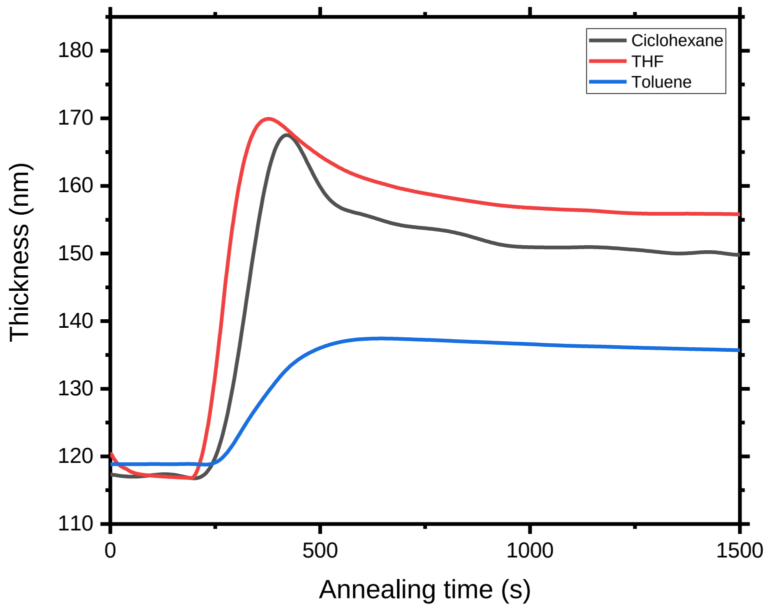

Figure S1. BCP film swelling during the SVA in cyclohexane (black line); THF (red line) and toluene (blue line) measured in-situ by ellipsometry.

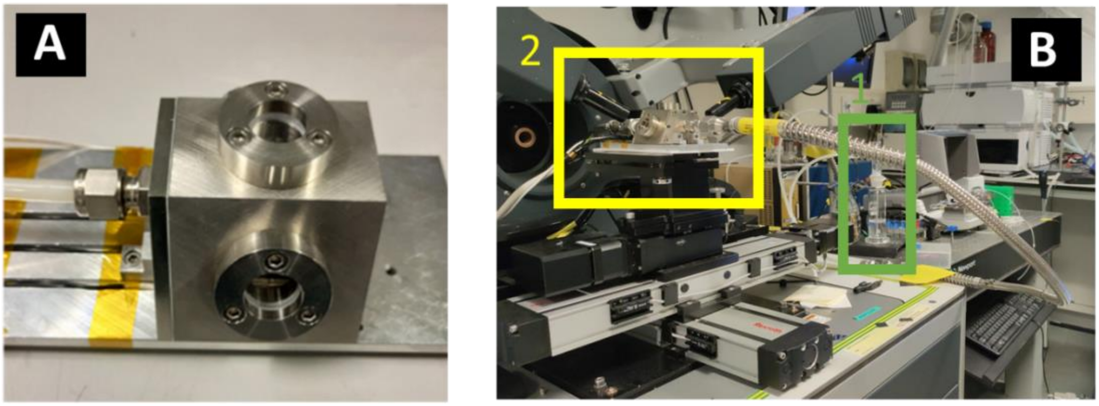

Figure S2. Photography of the SVA chamber used during this work (A). SVA experiment consist in a continuous gas flow passing through a reservoir containing cyclohexane (1B) which is then introduced in the chamber containing the hybrid film (2B). Film thickness was followed in situ by ellipsometry.

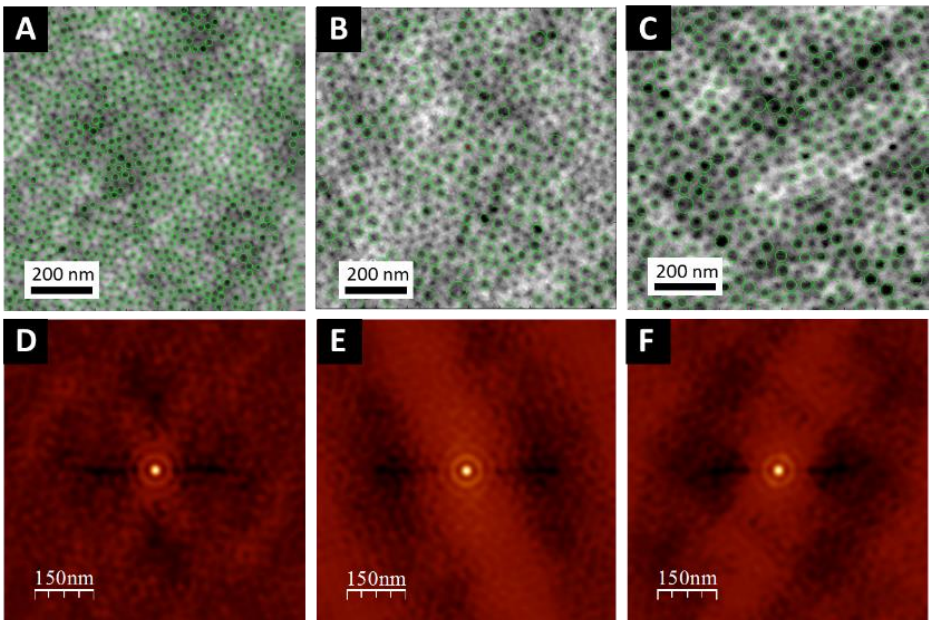

Figure S3. Image analysis of the AFM micrographs for pore size determination: A) no SVA; B) 30 min SVA and C) 1h SVA. D-F) 2D correlation images of the corresponding AFM images.

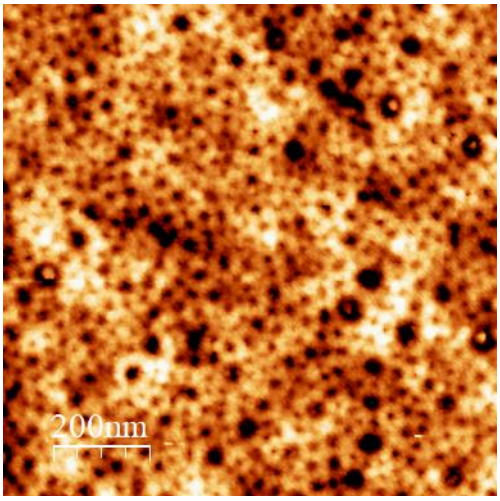

Figure S4. AFM topographical micrography of the TiO_2 inorganic mesoporous thin films obtained after 1.5 h under SVA.

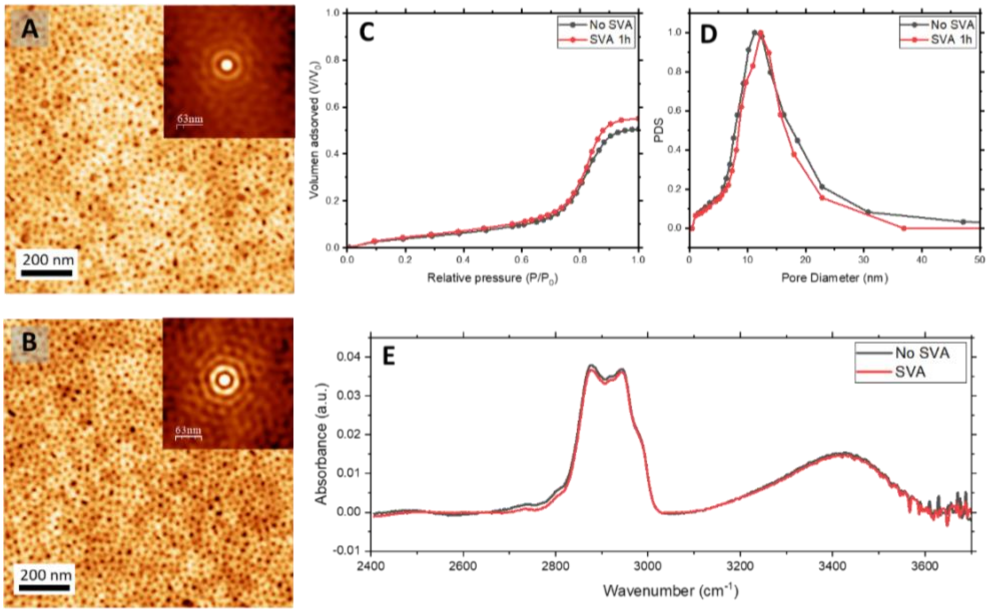

Figure S5. AFM topographical images of the aluminosilicate mesoporous films obtained with no SVA (A) and after 1h SVA treatment (B). EP adsorption isotherms (C) with correlated pore size distributions (D) of aluminosilicate mesoporous films obtained with no SVA (black line) and after 1h SVA treatment (red line). FTIR spectra of the hybrid aluminosilicate-BCP samples before (black line) and after (red and blue line) SVA (E).
